# RNA-Binding Proteins in the Control of LPS-Induced Macrophage Response

**DOI:** 10.3389/fgene.2019.00031

**Published:** 2019-02-04

**Authors:** Dirk H. Ostareck, Antje Ostareck-Lederer

**Affiliations:** Department of Intensive Care Medicine, University Hospital RWTH Aachen, Aachen, Germany

**Keywords:** RNA-binding proteins, post-transcriptional regulation, inflammation, bacterial lipopolysaccharides, macrophage activation

## Abstract

Innate immune response is triggered by pathogen components, like lipopolysaccharides (LPS) of gram-negative bacteria. LPS initiates Toll-like receptor 4 (TLR4) signaling, which involves mitogen activated protein kinases (MAPK) and nuclear factor kappa B (NFκB) in different pathway branches and ultimately induces inflammatory cytokine and chemokine expression, macrophage migration and phagocytosis. Timely gene transcription and post-transcriptional control of gene expression confer the adequate synthesis of signaling molecules. As *trans*-acting factors RNA binding proteins (RBPs) contribute significantly to the surveillance of gene expression. RBPs are involved in the regulation of mRNA processing, localization, stability and translation. Thereby they enable rapid cellular responses to inflammatory mediators and facilitate a coordinated systemic immune response. Specific RBP binding to conserved sequence motifs in their target mRNAs is mediated by RNA binding domains, like Zink-finger domains, RNA recognition motifs (RRM), and hnRNP K homology domains (KH), often arranged in modular arrays. In this review, we focus on RBPs Tristetraprolin (TTP), human antigen R (HUR), T-cell intracellular antigen 1 related protein (TIAR), and heterogeneous ribonuclear protein K (hnRNP K) in LPS induced macrophages as primary responding immune cells. We discuss recent experiments employing RNA immunoprecipitation and microarray analysis (RIP-Chip) and newly developed individual-nucleotide resolution crosslinking and immunoprecipitation (iCLIP), photoactivatable ribonucleoside-enhanced crosslinking (PAR-iCLIP) and RNA sequencing techniques (RNA-Seq). The global mRNA interaction profile analysis of TTP, HUR, TIAR, and hnRNP K exhibited valuable information about the post-transcriptional control of inflammation related gene expression with a broad impact on intracellular signaling and temporal cytokine expression.

## Introduction

The immune responses against bacteria, viruses and parasites require tight regulation, because uncontrolled, excessive or persisting immune reactions provoke inflammatory diseases (Zanotti et al., 2002). As a central component of the innate immune response, macrophages sense pathogen components such as lipopolysaccharides (LPS), an essential constituent of the outer membrane of gram-negative bacteria. Recognition of LPS by TLR4 on the macrophage surface results in the activation of MAPK and NFκB dependent signaling pathways, which activate inflammation related genes encoding pro- and anti-inflammatory cytokines and chemokines (Medzhitov and Horng, 2009; Takeuchi and Akira, 2010; Smale, 2012; Vaure and Liu, 2014). The underlying genome-wide changes in macrophage gene expression (Reynier et al., 2012; Rutledge et al., 2012) require downstream post-transcriptional checkpoints, which are critical for the appropriate modulation of immune reactions (Carpenter et al., 2014; Kafasla et al., 2014). Emerging experimental evidence highlights the impact of RNA binding proteins (RBPs) on the post-transcriptional control of the immune response (Fu and Blackshear, 2017; Garcia-Maurino et al., 2017; Diaz-Munoz and Turner, 2018; Mino and Takeuchi, 2018; Turner and Diaz-Munoz, 2018).

By analyzing RNA-protein interaction profiling and RNA sequencing experiments with TTP, HUR, TIAR, and hnRNP K we provide an overview on their target mRNAs, which are regulated at the level of mRNA stability and translation in LPS activated macrophages.

## Zink-Finger Protein TTP Controls Target mRNA Decay in Inflammation

**FIGURE 1:**
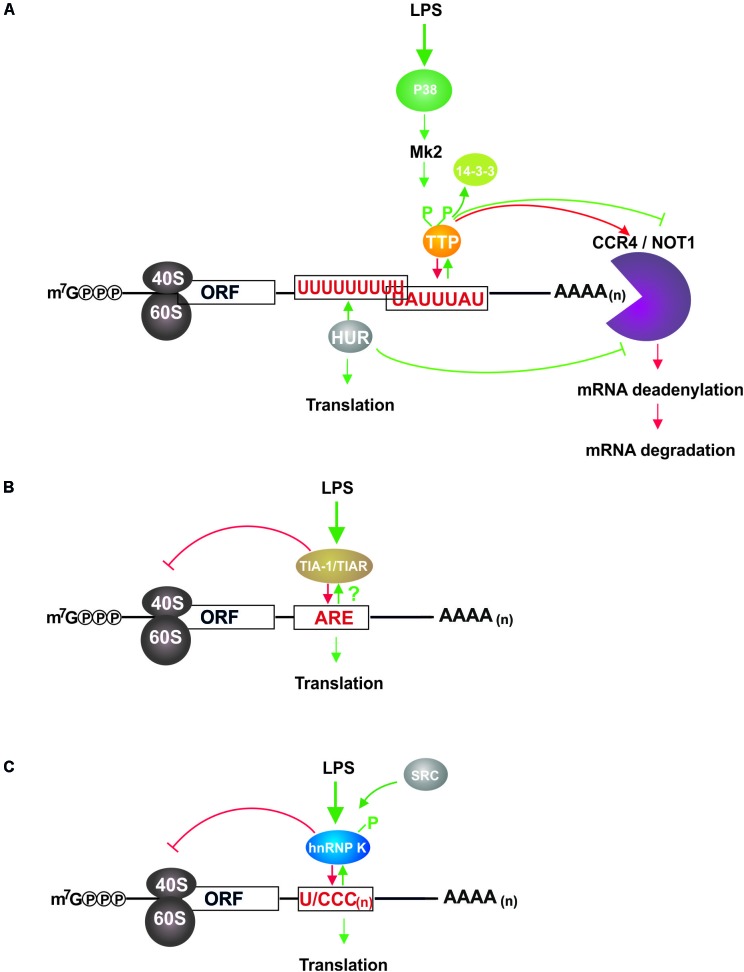
LPS induced impact of TTP, HUR, TIAR, and hnRNP K on mRNAs in macrophages. **(A)** TTP and HUR interact with U-rich elements in the 3′UTR of target mRNAs and regulate their stability and translation (Stoecklin et al., 2008; Tiedje et al., 2012, 2016; Sedlyarov et al., 2016). In non-induced cells bound TTP recruits the CCR4/NOT1 complex, which initiates 3′UTR deadenylation and 3′ to 5′ mRNA decay. LPS induced TLR4 signaling activates MK2, which phosphorylates TTP leading to its sequestration by 14-3-3 proteins and subsequent abolition of deadenylation complex association. Following T^118^ phosphorylation by MK2 HUR accumulates in the cytoplasm where it mediates mRNA stabilization, disables decay complex formation and enhances translation. By controlling mRNA decay, TTP curtails the synthesis of inflammation related proteins in non-induced macrophages, and regulates their balanced expression in response to LPS conjointly with HUR. **(B)** TIAR binding to AREs causes translational repression of target mRNAs, which is diminished by a so far unknown mechanism in LPS activated macrophages, resulting in elevated synthesis of cytokines (Piecyk et al., 2000; Kharraz et al., 2016). TIAR dampens the expression of immune response associated proteins, which can be activated immediately in response to LPS. **(C)** HnRNP K bound to U/CCC_(n)_ elements in the mRNA 3′UTR inhibits target mRNA translation, but is released from the binding site following c-Src catalyzed tyrosine phosphorylation that is initiated in response to LPS dependent macrophage activation (Liepelt et al., 2014). Thereby a rapid LPS response facilitated by straight signaling molecule synthesis can be established.

Tristetraprolin, encoded by the gene *Zfp36*, has been characterized as critical mRNA destabilizing protein in immune cells (Blackshear, 2002; Brooks and Blackshear, 2013). To initiate target mRNA decay, TTP mediates the recruitment of deadenylation and decapping complexes to the mRNA 3′ untranslated region (3′UTR) and 5′UTR, respectively (Fenger-Gron et al., 2005; Fabian et al., 2013). Tandem CCCH-type zinc-finger domains of TTP interact with AU-rich elements (ARE) that are mainly located in mRNA 3′UTRs (Lai et al., 1999; Worthington et al., 2002). In macrophages, target mRNAs primarily encode proteins related to inflammation response, among them cytokines and chemokines (Carballo et al., 1998; Lai et al., 1999; Stoecklin et al., 2008; Kratochvill et al., 2011; Sedlyarov et al., 2016; Tiedje et al., 2016). Under steady state conditions TTP is ubiquitously expressed at a basal level. Inflammatory stimuli like LPS and cytokines mediate transcriptional and post-transcriptional induction of TTP expression (Mahtani et al., 2001; Schaljo et al., 2009; Sedlyarov et al., 2016). AREs in TTP mRNA 3′UTR represent bona fide functional TTP binding sites. An auto-inhibitory feedback regulation established through the interaction of TTP with these AREs secures a decrease in TTP expression when inflammatory stimuli decline. Thereby TTP contributes to regulatory circuits, which prevent the development of chronic inflammation (Tchen et al., 2004; Schott et al., 2014). TTP deficiency in mice causes a systemic inflammatory syndrome, which is in part attributable to the absence of TTP-controlled tumor necrosis factor (TNFα) mRNA destabilization (Taylor et al., 1996; Carballo et al., 1997). In macrophages, LPS triggered TLR4 signaling leads to the stabilization of TTP target mRNAs and their enhanced translation (Tiedje et al., 2012, 2016). TTP serine phosphorylation catalyzed by TLR4 downstream kinase MK2 induces its sequestration by 14-3-3 proteins and target mRNA release (Chrestensen et al., 2004; Stoecklin et al., 2004). Hence CCR4-Not1 deadenylation complex recruitment is abrogated (Marchese et al., 2010; Clement et al., 2011; Sandler et al., 2011), target mRNAs are stabilized (Brook et al., 2006; Hitti et al., 2006) and translation-promoting factors replace TTP (Tiedje et al., 2012; Figure 1A).

Four studies examined the impact of TTP on inflammation-related pathways: (I) (Stoecklin et al., 2008) identified TTP bound mRNAs in untreated and LPS induced murine RAW264.7 macrophages by TTP co-immunoprecipitation and RIP-Chip analysis (Table 1). (II) To investigate TTP-driven mRNA decay (Kratochvill et al., 2011) treated bone marrow derived macrophages (BMDM) with actinomycin D for different times prior to RIP-chip (Table 1). (III) Employing iCLIP (Tiedje et al., 2016), identified TTP bound mRNAs in LPS treated BMDM from mice expressing GFP-TTP or the non-MK2 substrate variant (Table 1). The impact of TTP phosphorylation on global mRNA stability and mRNA translation was examined integrating iCLIP, RNASeq and Ribosome profiling (RiboSeq) (Tiedje et al., 2016). (IV) To map mRNA binding sites of endogenous TTP precisely and to unveil its role in inflammation resolution (Sedlyarov et al., 2016) applied PAR-iCLIP and RNASeq in BMDM of TTP^(wt)^ and TTP^(-/-)^ mice exposed to LPS for different times (Table 1).

**Table 1:** Experimental strategies, tools and conditions employed in the studies discussed.

RBP	Study	Mouse genotype	Cell type	LPS	Analytical strategy	Antibody	Data deposition
**TTP**	Stoecklin et al., 2008		RAW264.7 mouse macrophages	2 h,10 ng/ml LPS, *E. coli* 026:B6 (Sigma)	RIP-Chip Affymetrix Mouse Genome 430 2.0 array covering 39.000 transcripts	Rabbit anti-TTP	doi: 10.1074/jbc.M709657200
	Kratochvill et al., 2011	C57BL/6 mice TTP^(wt)^ and TTP^(-/-)^	BMDM	3 h, 10 ng/ml LPS, *E. coli* 055:B5 (Sigma)	RIP-Chip Affymetrix Mouse Gene ST 1.0 array covering 28 853 genes	Rabbit anti-TTP (Schaljo et al., 2009)	http://www.ncbi.nlm.nih.gov/geo/acc. GSE28880.
	Tiedje et al., 2016	C57BL/6 TTP^(-/-)^ mice expr. GFP-TTP^(wt)^ and GFP-TTP^(S52,178A)^	BMDM	1 h, 1 μg/μl LPS	iCLIP	GFP-nanobodies	http://www.ncbi.nlm.nih.gov/geo/acc. GSE81250 TTP-atlas: http://ttp-atlas.univie.ac.at
	Sedlyarov et al., 2016	C57BL/6 mice TTP^(wt)^ and TTP^(ΔM)^	BMDM	3 h, 6 h, 10 ng/ml LPS (Sigma)	PAR-iCLIP	Rabbit anti-TTP (Kratochvill et al., 2011)	http://www.ncbi.nlm.nih.gov/geo/acc. GSE63468 TTP-atlas: http://ttp-atlas.univie.ac.at
**HUR**	Sedlyarov et al., 2016	C57BL/6 mice TTP^(wt)^ and TTP^(ΔM)^	BMDM	3 h, 6 h, 10 ng/ml LPS (Sigma)	PAR-iCLIP	HUR, clone 3A2 (Thermo Fisher Sci.)	http://www.ncbi.nlm.nih.gov/geo/acc. GSE63468 TTP-atlas: http://ttp-atlas.univie.ac.at
**TIAR**	Kharraz et al., 2016		RAW264.7, expr. TIAR- or TIAR-ΔRRM-FLAG	2 h, 100 ng/ml LPS, *E. coli* 0127:B8 (Sigma)	RIP-Chip Mouse Exonic Evidence Based Oligonucleotide (MEEBO) array covering 38.784 70mer probes (Stanford University, United States)	Anti-FLAG M2 agarose (Sigma)	http://www.ncbi.nlm.nih.gov/geo/acc.GSE77577
**hnRNP K**	Liepelt et al., 2014		RAW264.7 mouse macrophages	6 h, 10 ng/ml LPS, *E. coli* 0111:B4 (Sigma)	RIP-Chip Affymetrix Mouse Genome 430 2.0 array covering 39.000 transcripts	monoclonal hnRNP K (Naarmann et al., 2008)	http://www.ncbi.nlm.nih.gov/geo/acc.GSE48463


*Information related to experimental tools and conditions as provided in the original papers.*

Detailed inspection of sequence motifs in TTP bound mRNAs, which were detected in the different studies (Table 1) revealed AU-rich TTP binding sites represented by AUUUA pentamers and UUAUUUAUU nonamers (Stoecklin et al., 2008). In target mRNA 3′UTRs UAUUUAU heptamers (Sedlyarov et al., 2016) are highly enriched as well. MRNAs encoding checkpoint regulators of LPS induced inflammation response, e.g., TNFα, IL-10, IL-15, CXCL2, and CCL2 were identified with all applied experimental and data validation strategies. Interestingly Kratochvill et al. (2011), reported that 25% of LPS induced transcripts were unstable. Among those displaying a TTP dependent decay were TNFα, IL-6, IL-10, TTP, CXCL1, CXCL2, CSF2, IER3 encoding transcripts (Kratochvill et al., 2011), which were also identified in the study of Tiedje et al. (2016). These data support the hypothesis that TTP functions in the elimination of inflammation related mRNAs, the maintenance of a balanced LPS response and the resolution of inflammation. Related pathways corresponding to enriched mRNAs include TNFα-, NFκB-, Wnt- and chemokine signaling, the formation of focal adhesions, apoptosis and mRNA processing. They were as well covered by mRNAs detected in RiboSeq experiments (Tiedje et al., 2016). The top 25 mRNAs, which were differentially bound by GFP-TTP and GFP-TTP^(S52,178A)^ upon LPS stimulation include not only TNFα and NFκB-related signaling molecules like TNF, CXCL2, CXCL3, but also IER3 and DUSP1, which encode feedback inhibitors of the inflammatory response (Tiedje et al., 2016). These findings emphasize the importance of the MK2 dependent TTP release from target mRNAs to safeguard accurate feedback regulation of the inflammatory response. Remarkably, in the study of Sedlyarov et al. (2016) 343 TTP target mRNAs were identified through intron sequences bound by TTP. Of this group only 1% exhibited TTP dependent destabilization, suggesting that TTP binding to intron sequences does not affect intron processing. To characterize TTP impact on early and late LPS response (Sedlyarov et al., 2016) applied LPS treatment for 3 and 6 h. In the early phase only a few drivers of inflammation, such as TNFα, which activates central transcription inducers, e.g., NFκB are strongly controlled by TTP. In early and late response, the GO term coverage of target mRNA mostly overlapped. However, GO terms taxis and chemotaxis, which characterize the perpetuation of inflammation were only represented at the late response time point (Sedlyarov et al., 2016). From their analysis the authors conclude that TTP supports a switch to inflammation resolution by destabilizing mRNAs that encode migration-associated proteins, thereby impeding chronic inflammation.

## HUR a Versatile RRM Domain Protein Modulates mRNA Stability

The ubiquitously expressed protein HUR, which is encoded by *Elavl1*, consists of two consecutive N-terminal RNA recognition motifs (RRM), a central less conserved basic hinge region and a third C-terminal RRM (Ma et al., 1996). Whereas RRM1 and RRM2 function in RNA binding, RRM3 contributes to RNA-protein complex stabilization and protein-protein interactions, including HUR dimerization (Pabis et al., 2018). The basic hinge region includes a shuttling domain (Fan and Steitz, 1998), which in response to stress and mitogen signaling, facilitates nuclear-cytoplasmic shuttling of the predominantly nuclear HUR (Keene, 1999). Cytoplasmic HUR accumulation, which is induced by p38 and MK2 dependent T^118^ phosphorylation in response to γ-irradiation and oxidative stress, augments its binding to p21, urokinase and urokinase receptor mRNAs and their stabilization (Tran et al., 2003; Lafarga et al., 2009). HUR binds AREs, mostly located in the target mRNA 3′UTR (Fan and Steitz, 1998; Lopez de Silanes et al., 2004), but also in intron sequences. HUR binding can contribute to alternative pre-mRNA splicing for specific genes like ZNF207, GANAB, DST and PTBP2 (Lebedeva et al., 2011) and the differential stabilization of 3′UTR ARE containing c-Fos and c-Jun mRNAs (Peng et al., 1998). Systematic mapping and functional evaluation of HUR-RNA interactions by PAR-Clip and RIP-Chip experiments employing HEK293 cells confirmed that HUR mediates the modulation of nuclear pre-mRNA processing and stabilizes cytoplasmic mRNAs, which bear both intronic and 3′UTR binding sites (Mukherjee et al., 2011). Interestingly, as shown for miR-7 that is encoded in the last HNRNPK exon, HUR binding to specific intronic miRNA precursors is implicated in their processing. HUR depletion from HeLa cells results in upregulation of miR-7, whereas hnRNP K expression remains unaffected, suggesting that HUR controls miR-7 precursor processing (Lebedeva et al., 2011). Implementing a refined digestion optimized RIP-seq protocol (DO-RIP-seq) (Nicholson et al., 2017a,b) were able to quantify HUR binding sites transcriptome-wide. Since HUR target mRNAs encode proteins implicated in cell cycle control, cell death and differentiation, post-translational HUR modifications and dysregulated functions are associated with a broad range of pathologic conditions (Srikantan and Gorospe, 2012; Grammatikakis et al., 2017). Disease-linked HUR phosphorylation, methylation and proteolytic cleavage not only regulate the subcellular localization of HUR, but affect as well its RNA-binding (reviewed in Grammatikakis et al., 2017). Notably, in HeLa cells, fibroblasts and carcinoma tissues HUR controls the stability and interactions of lncRNAs, such as *HOTAIR* (Yoon et al., 2013), *LincRNA-p21* (Yoon et al., 2012), and *7SL* (Abdelmohsen et al., 2014) and adjusts their function in gene expression control. Furthermore, HUR and specific miRNAs cooperate or compete in mRNA regulation (Srikantan et al., 2012). Modulation of miRNA binding by HUR has been reported in human MCF-7 epithelial and Huh7 liver cells (Poria et al., 2016), as well as in murine BMDM (Lu et al., 2014). Remarkably, in BMDM LPS induced MK2 catalyzed TTP phosphorylation causes a shift of the competitive binding equilibrium between HUR and TTP toward HUR, which stabilizes TNFα mRNA and stimulates its translation (Tiedje et al., 2012; Figure 1A). This finding corroborates a functional relevance of a regulated crosstalk between HUR and TTP in the LPS induced macrophage immune response. In their comprehensive PAR-iCLIP and RNASeq analysis of the BMDM response to LPS (Sedlyarov et al., 2016) mapped HUR and TTP mRNA binding sites comparatively. The study revealed that a UUUUUUUUU nonamer is the most overrepresented HUR binding motif. With 78% the majority of HUR binding sites was located in 3′UTRs, which exceeds two times the number of TTP 3′UTR sites, whereas in intron sequences only 17% of the HUR sites were identified. Binding sites for both, TTP and HUR were determined in 59 target mRNAs. 552 and 120 binding sites for HUR and TTP, respectively, were not overlapping and 118 sites did overlap by at least 1 nt (Sedlyarov et al., 2016). This overlap applied to 40 targets, including TNFα and CXCL2 mRNA, for which simultaneous TTP and HUR binding were confirmed experimentally. Stability and expression of mRNAs bearing solely TTP binding sites did not significantly differ from mRNAs with overlapping motifs, suggesting no co-regulation of mRNA stability (Sedlyarov et al., 2016) in macrophage inflammatory response, but possibly at the level of mRNA translation as shown for TNFα mRNA (Tiedje et al., 2012).

## TIAR, a RRM Domain Protein Contributes to mRNA Translation Control

The two closely related DNA/RNA-binding proteins, T-cell intracellular antigen 1 (TIA-1) (Anderson et al., 1990) and TIA-1 related protein (TIAR), contain three N-terminal RRMs, which mediate oligonucleotide binding and a C-terminal Q-rich prion-related domain that enables participation in stress granule formation (Waris et al., 2014). TIAR RRM1 preferentially interacts with T-rich ssDNA and functions in transcription activation (Suswam et al., 2005). RRM2 displays affinity for U- and RRM3 for C-rich motifs (Dember et al., 1996; Cruz-Gallardo et al., 2014), whereas the RRM23-tandem domain binds mainly UC-rich sequences (Waris et al., 2017).

RRM2 and RRM3 contribute to nuclear accumulation of TIA proteins and nuclear export, respectively (Zhang et al., 2005). Both interact with U-rich stretches near mRNA 5′-splice sites (Del Gatto-Konczak et al., 2000) and modulate alternative splicing of mRNAs encoding FAS in murine fibroblasts (Forch et al., 2000), NF1 in rat neuronal cells (Zhu et al., 2008), human chondrocyte COL2A1 (McAlinden et al., 2007), liver CFTR (Zuccato et al., 2004), and CGRP in HeLa cells (Zhu et al., 2003). Furthermore, TIA proteins control TIAR and TIA-1 isoform expression tissue- and cell type specific (Izquierdo and Valcarcel, 2007).

In the cytoplasm, TIA proteins interact with 3′UTR AREs of mRNAs encoding inflammation related proteins. TIAR was shown to bind to the TNFα mRNA 3′UTR in RAW264.7 cells (Lewis et al., 1998; Gueydan et al., 1999) and peritoneal macrophages (Piecyk et al., 2000) in an LPS dependent manner. Enhanced TNFα synthesis in macrophages of TIAR^(-/-)^ mice (Piecyk et al., 2000) suggests that in unstimulated cells TIAR impedes TNFα mRNA translation, which can be activated to drive inflammatory cytokine expression upon TLR4 mediated recognition of bacterial LPS (Figure 1B). TIA protein mediated control of TNFα expression is demonstrated by impaired TNFα mRNA regulation in TNFα^(ΔARE)^ mice, where it is implicated in chronic inflammation (Kontoyiannis et al., 1999). Furthermore, mRNAs encoding inflammation related COX-2 (Cok et al., 2003; Dixon et al., 2003) and HMMP-13 mRNA (Yu et al., 2003) are TIAR targets in primary murine fibroblasts and human mesangial cells, respectively.

Interestingly, TIA proteins have also been shown to contribute to global translation regulation under amino acid starvation in HEK293S cells. TIA proteins bind to the 5′-oligopyrimidine tract of 5′-TOP mRNAs, which encode critical components of the translational apparatus, like ribosomal proteins and PABP-C1 and induce the release of these target mRNAs from actively translating polysomes (Damgaard and Lykke-Andersen, 2011). Besides that, TIA proteins are involved in the formation of stress granules, which sequester mRNAs that are translationally stalled by specific mRNPs under starvation-induced stress (Damgaard and Lykke-Andersen, 2011), heat shock and arsenide stress in fibroblasts (Kedersha et al., 1999), in LPS activated B-cells (Diaz-Munoz et al., 2017) and other adverse conditions including hypoxia and viral infection (Anderson and Kedersha, 2002; Waris et al., 2014).

RIP-Chip experiments were performed by Kharraz et al. (2016) to identify mRNAs specifically bound by TIAR in unstimulated and LPS induced murine RAW264.7 macrophages stably expressing TIAR^(wt)^-FLAG and TIAR^(ΔRRM2)^-FLAG. RRM2 of TIAR, which is required for high affinity mRNA binding (Dember et al., 1996; Kim et al., 2013) was deleted to discard all mRNAs that bind with low affinity. The analysis revealed that 351 mRNAs were bound by TIAR in unstimulated macrophages and 779 in LPS induced cells, with 8 transcripts exclusively bound in unstimulated and 436 in LPS induced cells, respectively. Binding of TNFα mRNA could be validated, also the binding of the mRNA that encodes MAPK phosphatase 1 (MKP-1 also termed CL100, VHV1, 3CH134, and DUSP1), for which an interaction with TIAR has been shown before in HeLa cells (Kuwano et al., 2008). The mRNAs encoding TLR4 and the serine/threonine phosphatase 2A catalytic subunit 2β could be identified as new targets (Kharraz et al., 2016). GO term analysis of TIAR target mRNAs shows that under both condition the GO terms catabolic process, cell cycle and regulation of apoptosis were enriched, which cover proteins involved in inflammatory response, cell proliferation, cell death and metabolism. However, exclusively in LPS induced cells mRNAs bound by TIAR encoded proteins within the GO term category positive regulation of IkB kinase/NFκB cascade (Kharraz et al., 2016).

T-cell intracellular antigen 1 related protein ARE binding specificity was lower than that of TTP (Stoecklin et al., 2008), but not affected by LPS treatment (Kharraz et al., 2016). The high number of mRNAs bound in response to LPS suggests that LPS directly modulates TIAR mRNA binding and that TIAR interacts, mediated by RRM1 and RRM3, with additional ARE-independent sequence motifs (Kharraz et al., 2016). TIAR mediated regulation of alternative mRNA splicing and inhibition of mRNA translation, which were shown for inflammation related proteins (Gueydan et al., 1999; Piecyk et al., 2000; Cok et al., 2003; Suswam et al., 2005), indicate that TIAR modulates the inflammatory response and contributes to its rapid decline when the stimulus disappears.

## KH Domain Protein hnRNP K Modulates mRNA Translation

Heterogeneous ribonuclear protein K was first described as a structural component of nuclear ribonucleoprotein complexes associated with heterogeneous nuclear RNA (Pinol-Roma et al., 1988; Matunis et al., 1992). The protein contains three KH domains consisting of 65–70 amino acids (Gibson et al., 1993; Siomi et al., 1993; Dejgaard and Leffers, 1996), which occur with two distinct folding topologies (Baber et al., 1999; Grishin, 2001). In SELEX experiments UC_3-4_ RNA motifs were determined as optimal ligands for KH3 (Thisted et al., 2001). Binding of hnRNP K KH domains to ssDNA (Braddock et al., 2002; Backe et al., 2005) and RNA (Messias et al., 2006; Moritz et al., 2014) has been analyzed systematically. Quantitative evaluation indicated that the KH domains of hnRNP K contribute differentially to RNA binding, with KH1-KH2 acting as a tandem domain and KH3 as an individual binding domain (Moritz et al., 2014). The affinity of full-length hnRNP K is in the nanomolar range, while K_D_ values for the isolated domain KH3 are micromolar (Backe et al., 2005; Moritz et al., 2014). Two U/CCC motifs within 19 nts confer hnRNP K binding, whereas four U/CCC motifs within 38 nts are necessary and sufficient for translational regulation (Ostareck et al., 1997). Bidirectional nuclear-cytoplasmic transport of hnRNP K is mediated by an N-terminal nuclear localization motif and a hnRNP K-specific shuttling domain (Michael et al., 1995, 1997).

As multifunctional protein hnRNP K is associated with transcription activation (Moumen et al., 2005), pre-mRNA splicing (Expert-Bezancon et al., 2002), mRNA stability control (Skalweit et al., 2003) and regulation of mRNA translation (Ostareck et al., 1997, 2001; Collier et al., 1998; Naarmann et al., 2008, 2010; Naarmann-de Vries et al., 2016). HnRNP K functions are modulated by mRNA specific associated mRNP components (Ostareck-Lederer and Ostareck, 2012) and by post-translational modifications. ERK dependent phosphorylation of S^284,353^ drives its cytoplasmic accumulation as prerequisite for hnRNP K-mediated mRNA translation regulation (Habelhah et al., 2001). Phosphorylation of KH3 Y^458^ by c-Src (Ostareck-Lederer et al., 2002; Messias et al., 2006; Adolph et al., 2007) and caspase-3 catalyzed cleavage (Naarmann-de Vries et al., 2013) control hnRNP K release from translational regulated target mRNAs and site-specific arginine methylation by PRMT1 regulates hnRNP K protein-protein interactions (Ostareck-Lederer et al., 2006).

In human Thp-1 monocytes, hnRNP K was shown to be associated with the COX-2 promoter and to control cytoplasmic COX-2 mRNA stability by modulating miRNA binding to the 3′UTR (Shanmugam et al., 2008).

RIP-Chip analysis of differential mRNA binding in untreated and LPS induced RAW264.7 macrophages demonstrated that 1901 mRNAs were differential bound by hnRNP K in response to LPS. GO term annotation allocated them to biological processes related to metabolism, cell communication, transport, cell cycle, development and immune response (Liepelt et al., 2014). Strikingly, whereas cytokines and chemokines were underrepresented among the 163 mRNAs related to immune response, 21 mRNAs encoded kinases and modulators in TLR4 signaling, of which 7 equally expressed mRNAs encoding IRAK4, IRAK1BP1, ERC1, CARM1/PRMT4, PI3KCA, AKT3, and TAK1 were specifically enriched in non-induced cells (Liepelt et al., 2014). A detailed analysis of differential hnRNP K association with the mRNA of transforming growth factor-ß-activated kinase 1 (TAK1), a central kinase in TLR4 signaling, revealed that KH domain 3 interacts with U/CCC elements in the TAK1 mRNA 3′UTR. Silencing of hnRNP K expression in macrophages and BMDM had no impact on the level of TAK1 mRNA, but endogenous TAK1 mRNA accumulated in actively translating polysomal fractions, resulting in an increased TAK1 expression. Through the regulation of TAK1 mRNA translation hnRNP K affects the phosphorylation of the TAK1 downstream target p38 and finally inflammatory cytokine gene transcription, i.e., TNFα, IL-1ß, and IL-10 (Liepelt et al., 2014), thereby hnRNP K modulates the LPS response of macrophages (Figure 1C).

## Conclusion and Perspectives

As primary responding cells of the innate immune system, macrophages recognize pathogens and become activated to initiate and coordinate the organism-wide systemic immune response by cytokine and chemokine secretion, migration and phagocytosis. These processes require highly coordinated gene expression, which is achieved at the post-transcriptional level by regulated functional RBP-RNA interactions. Specific RBPs, TTP, HUR, TIAR, and hnRNP K, regulate the fate of their cellular target RNAs from synthesis to turnover and translation, thereby contributing to the coordination of the rapid and purposeful immune cell responses. LPS molecules of gram-negative bacteria are abundant and specific ligands that activate macrophages through TLR4 receptor signaling. Systematic analyses of RBP-RNA interaction in untreated and LPS-induced macrophages employing RIP-Chip, iCLIP, PAR-iCLIP, RNAseq, and RiboSeq studies revealed a first insight in the complex protein-mRNA networks that are established by RBPs, which bind mRNAs with different specificities for AREs and U-rich elements, like TTP, HUR, and TIAR and pyrimidine-rich sequence motifs, like hnRNP K based on their individual domain composition. These specific interactions ensure the simultaneous modulation of various target mRNAs, which encode proteins that function in defined biological processes related to the induction and resolution of immune response. Whereas TIAR and hnRNP K mainly modulate mRNA translation, which enables the direct regulation of signaling protein synthesis to initiate immune response, TTP and HUR are primarily involved in the control of mRNA decay and stability that is required to balance and resolve immune reactions.

These processes have been studied so far only for a limited number of mRNAs differentially bound by RBPs in response to LPS. It will be essential and informative to investigate the regulation of further target mRNAs of TTP, HUR, TIAR, and hnRNP K discovered in these studies to get more insight in regulatory feedback mechanisms that coordinate the balanced immune response and its dysregulation in chronic inflammation.

To this end it is interesting to note that a number of unconventional RBPs, have been identified recently, for which RNA related functions that will expand our understanding of post-transcriptional gene regulation still need to be elucidated (Castello et al., 2015; Albihlal and Gerber, 2018; Hentze et al., 2018).

In macrophages, 19 new putative RBPs, which lack well characterized RNA binding domains were identified by RNA interactome capture (Liepelt et al., 2016). Panther protein class annotation revealed that they are involved in signaling, enzymatic functions and cytoskeletal remodeling. It will be interesting to identify their target mRNAs and to study their potential functions in LPS induced macrophage response.

In addition, post-transcriptional RNA modifications (Nachtergaele and He, 2018) might add a further layer of regulation in LPS-induced macrophages, affecting RBP binding and thereby the fate of their target mRNAs.

## Author Contributions

All authors listed have made a substantial, direct and intellectual contribution to the work, and approved it for publication.

## Conflict of Interest Statement

The authors declare that the research was conducted in the absence of any commercial or financial relationships that could be construed as a potential conflict of interest.
